# Impact of telehealth interventions added to peritoneal dialysis-care: a systematic review

**DOI:** 10.1186/s12882-022-02869-6

**Published:** 2022-08-23

**Authors:** Geertje K. M. Biebuyck, Aegida Neradova, Carola W. H. de Fijter, Lily Jakulj

**Affiliations:** 1 Dianet Dialysis Center/Division of Nephrology, Department of Medicine, Amsterdam UMC location University of Amsterdam, Meibergdreef 9, AZ 1105 Amsterdam, the Netherlands; 2 Department of Medicine, Division of Nephrology, Amsterdam UMC location University of Amsterdam, Internal Medicine and Nephrology, Meibergdreef 9, AZ 1105 Amsterdam, the Netherlands; 3 Department of Internal Medicine and Nephrology OLVG, Amsterdam, the Netherlands

**Keywords:** Telehealth, E-health, Telemedicine, Peritoneal dialysis, Home-dialysis, Covid-19

## Abstract

**Background:**

Telehealth could potentially increase independency and autonomy of patients treated with peritoneal dialysis (PD). Moreover, it might improve clinical and economic outcomes. The demand for telehealth modalities accelerated significantly in the recent COVID-19 pandemic. We evaluated current literature on the impact of telehealth interventions added to PD-care on quality of life (QoL), clinical outcomes and cost-effectiveness.

**Methods:**

An electronic search was performed in Embase, PubMed and the Cochrane Library in order to find studies investigating associations between telehealth interventions and: i. QoL, including patient satisfaction; ii. Standardized Outcomes in Nephrology (SONG)-PD clinical outcomes: PD-related infections, mortality, cardiovascular disease and transfer to hemodialysis (HD); iii. Cost-effectiveness. Studies investigating hospitalizations and healthcare resource utilization were also included as secondary outcomes. Due to the heterogeneity of studies, a meta-analysis could not be performed.

**Results:**

Sixteen reports (*N* = 10,373) were included. Studies varied in terms of: sample size; design; risk of bias, telehealth-intervention and duration; follow-up time; outcomes and assessment tools. Remote patient monitoring (RPM) was the most frequently studied intervention (11 reports; *N* = 4982). Telehealth interventions added to PD-care, and RPM in particular, might reduce transfer to HD, hospitalization rate and length, as well as the number of in-person visits. It may also improve patient satisfaction.

**Conclusion:**

There is a need for adequately powered prospective studies to determine which telehealth-modalities might confer clinical and economic benefit to the PD-community.

**Supplementary Information:**

The online version contains supplementary material available at 10.1186/s12882-022-02869-6.

## Introduction

In Europe, approximately 250,000 patients depend on dialysis for their survival. This number is increasing by 5–8% per year, due to ageing and the rising incidence of diabetes mellitus and hypertension [1]. Peritoneal dialysis (PD) is a home-based dialysis treatment, carried out autonomously by the patient or with the assistance of an informal or professional caregiver. PD provides more flexibility to patients, improves health-related quality of life (QoL), with similar clinical outcomes and survival as compared to in-center hemodialysis (ICHD) [2–4]. Moreover, of the distinct dialysis modalities, PD confers the lowest (non)-dialysis-related costs [5, 6]. Hence, an increased number of patients opting for PD could strongly reduce the high resource and budget impact of dialysis treatment on national healthcare systems [5, 6]. Despite these potential advantages for patient and society, merely 20% of the Dutch patients starting with dialysis, start with PD [7]. PD utilization is even lower in other parts of the world [8–10]. A potential drawback for both patients and professionals is that PD requires a certain level of treatment-specific education, as well as an active attitude from the patient, partner or caregiver. In addition, the lack of ability for the healthcare team to monitor the treatment real-time and to intervene when necessary, may contribute to the reserve that patients and clinicians have against engaging in a home-based dialysis treatment [11–13]. E-health interventions allowing for bi-directional data exchange and communication between patient and healthcare team could support patients in their home-dialysis treatment by facilitating education about home-dialysis, self-management and thereby increase feelings of safety. In addition, telehealth could allow the healthcare team to timely discover trends in relevant treatment-related data, which precede possible unfavorable clinical outcomes such as fluid overload, infections, hospitalizations or technique failure (i.e. the need to switch from PD to HD). Although remote patient monitoring (RPM) is gaining ground in automated PD (APD), in current continuous ambulant PD (CAPD) management, treatment-related data are mostly collected on paper by the patient accompanied by communication by telephone with the healthcare team or at the outpatient clinic. This is in great contrast with the use of digital monitoring and smartphone apps in almost all aspects of daily life nowadays.

Despite the growing interest in the use of e-health-based interventions in home-dialysis, both the reported interventions and studied outcomes are heterogeneous, thereby limiting evidence regarding effectiveness in terms of improvement of standardized clinical outcomes and associated impact on healthcare efficiency and economics [14]. Recently, the number of e-health initiatives and publications amplified, largely accelerated by the COVID-19 pandemic, resulting in seven new studies on this topic, representing 9377 patients receiving PD [15–21]. Furthermore, the importance of home-dialysis and telemedicine support in the recent COVID-19 pandemic has recently been underlined by the ERA-EDTA Working Group and by ISPD [22]. Hence, due to this substantial increase in the number of publications on the topic, as well as the increased urgency for the utilization and optimization of PD as a home-dialysis treatment, we performed a contemporary systematic review aimed to study the impact of telehealth interventions added to PD care in terms of QoL, Standardized Outcomes in Nephrology (SONG)-PD clinical outcomes [23] and cost-effectiveness.

## Methods

### Search strategy

An electronic search strategy was performed in Embase, Pubmed and the Cochrane Library to find eligible reports from January 1st 2010 until to March 1st 2021. The following terms were used: ‘*peritoneal dialysis*’, ‘*intermittent peritoneal dialysis*’, ‘*peritoneum dialysis*’, ‘*telemonitoring*’, ‘*distant (patient) monitoring*’, ‘*remote (patient) monitoring*’, *‘telemedicine’*, ‘*telehealth*’, ‘*e-health*’, *‘cell phone’, ‘tablets’, ‘device’, ‘smart phone’, ‘virtual consultation’, ‘video consultation’, ‘remote treatment monitoring’*. Synonyms of all terms were added in this search strategy (Supplementary Material I). Titles and abstracts were reviewed by two reviewers (GB and LJ), with consultation of a third reviewer in case of doubt (AN). The full-text screening of publications, including the reference lists, in order to identify possible additional eligible studies was performed by the same two reviewers (GB and LJ). No review protocol was made for this systematic review.

### Eligibility criteria and outcome measures

We included studies according to the following criteria: adult patients treated with peritoneal dialysis (APD or CAPD); implementation of any form of tele-monitoring, telemedicine or e-health that meets the definition of the World Health Organization [24] and assessment of any of the following as primary outcomes: i. quality of life; ii. any of the SONG-PD clinical outcomes [23]: PD-related infections, mortality, cardiovascular disease or technique failure (defined as transfer to HD); iii. Cost-effectiveness. Studies investigating hospitalization rates or healthcare resource consumption, i.e. length of hospitalization and the frequency of (in person) consultations as primary outcomes were included as secondary outcomes in our current systematic review and analysis.

There were no restrictions regarding experimental study design or methodology, except for the exclusion of simulation-studies not involving actual patients. Case reports, conference abstracts, reviews and perspectives were also excluded, as well as publications in any other language than English, Dutch or French.

### Data-extraction and analysis

Data extraction and quality assessment was performed using the Cochrane Risk of Bias assessment tool for randomized studies (version 2011) [25] and the ROBINS-I tool for non-randomized studies (version 2016) [26], respectively. Risk of bias was assessed by two reviewers (GB and LJ) using these tools. A third reviewer was consulted (AN) in case of doubt.

Since a meta-analysis was not possible for any of the outcomes, a descriptive evaluation of primary and secondary outcomes was conducted by clustering reports according to the investigated outcome of interest. Results of this systematic review were reported according to the PRISMA 2020 statement [27].

## Results

**Fig. 1:**
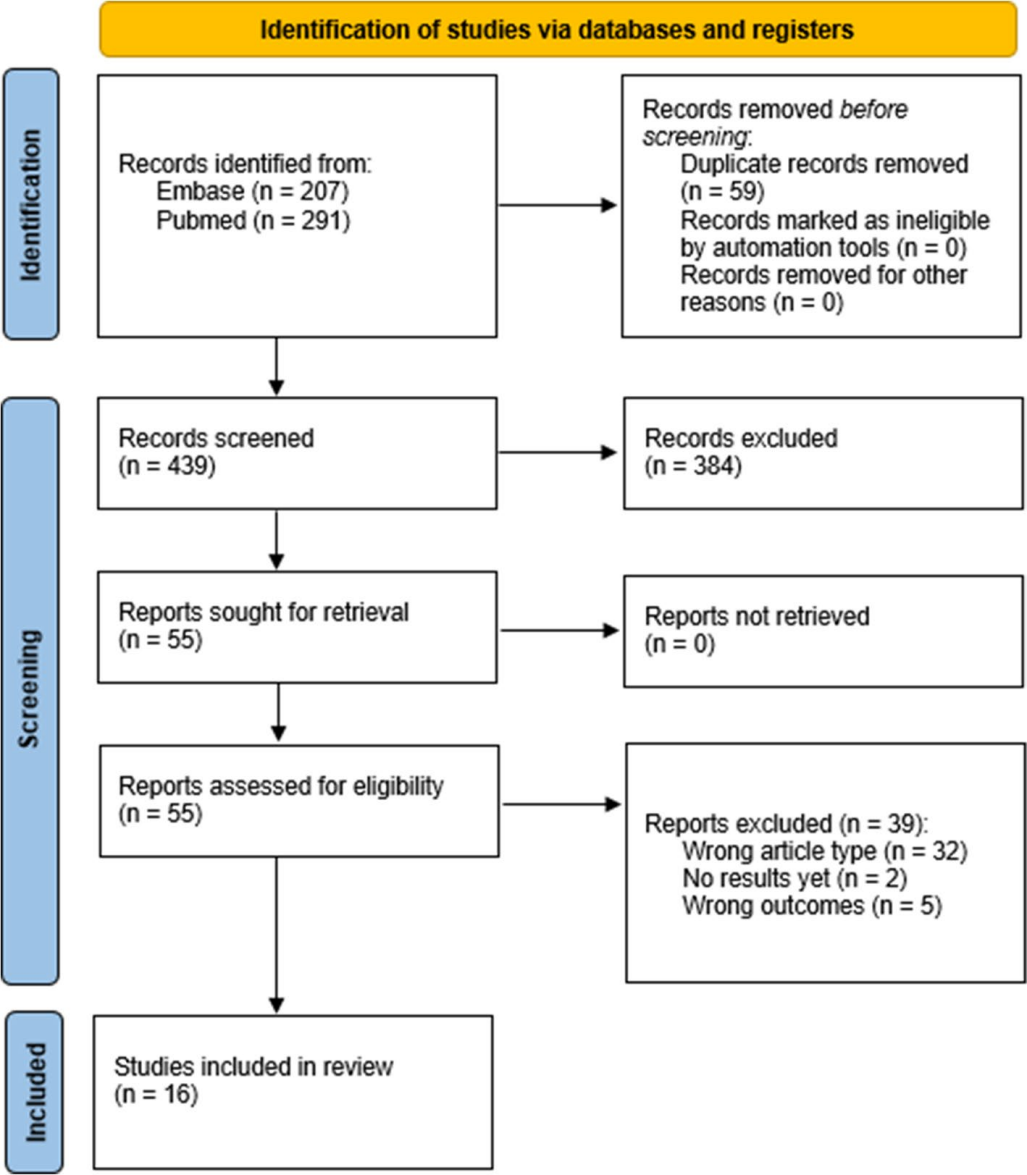
PRISMA 2020 flow diagram of included studies

The search strategy yielded 439 publications to be screened. Of these, fifty-five full-text articles were extracted and reviewed. Finally, sixteen reports met all eligibility criteria for inclusion in the systematic review (Fig. 1).

### Study and patient characteristics

We included sixteen studies in the review [15–21, 28–36]. Together, these studies represent 10,373 patients treated with PD, ranging from *N* = 6 to *N* = 6434. At least 11.8% (*N* = 1222) of these patients were treated with CAPD [15, 16, 28–30]. Five studies did not specify the PD-treatment modality of the participants [17, 18, 31–33]. Approximately 40% of the participants were female. The average age of participants was 57.3 ± 5.5 years. One study did not report the mean age of the study group [33]. The mean duration of patient follow-up was 181 ± 571 months. Two studies did not report the duration of follow-up [15, 33]. Table 1 displays characteristics of the included studies stratified by the studied outcomes of interest. These studies include two randomized controlled trials [28, 30], one prospective cohort study [16], four observational cohort studies [17–19, 33], five retrospective cohort studies [20, 21, 31, 34, 36] and four pilot studies [15, 29, 32, 35]. Four studies were conducted in the United States of America [15, 29, 31, 33], three were performed in Italy [18, 20, 21], three in Colombia [19, 34, 36], two in China [28, 30] and one in the Dominican Republic [16], India [17], the United Kingdom [35] and Canada [32], respectively.

**Table 1:** Characteristics of included studies stratified by studied outcomes

Study	Country	Population	Study design	Intervention	Comparison	Follow-up	Outcomes	Results	Risk of bias
Cao 2018 [28]	China	*N* = 160 Age 52.2 ± 15y M = 58% CAPD	RCT	Internet-based instant messaging software (N = 80)	Traditional follow-up (N = 80)	11.4 ± 1.5 months	Patient-satisfaction [modified from] [37]	Higher in the intervention group (*p* < 0.001, 98.1% vs 92.1%)	Unclear
							Mortality	Lower in intervention group (p = 0.058, number of events not reported)	
							Exit-site infection	N.S. difference	
							Peritonitis	Higher in intervention group (60 cases in 80 patients (75%) vs 40 cases in 80 patients (50%) statistical significance not reported)	
							Transfer to HD (was not a pre-specified outcome)	N.S. difference	
							Hospitalizations	N.S. difference	
Li 2014 [30]	China	*N* = 135 Age 56.3 ± 12.4y M = 59% CAPD	RCT	Post-discharge nurse-led telephone support (*N* = 69)	Routine hospital discharge care (*N* = 66)	12 weeks	QoL (KDQOL-SF)	N.S. difference	Unclear
							Patient satisfaction (sub-item of KDQOL-SF)	Higher in intervention group (p < 0.01, 73.7% vs 70.5%)	
							Peritonitis	N.S. difference	
							Catheter-infections	N.S. difference (data not shown)	
							Readmissions	N.S. difference	
							Clinical visits	Less in intervention group (71% vs 47%, *p* = 0.039)	
Sanabria 2019 [36]	Colombia	*N* = 360 Age 57 ± 17y M = 56% APD incident patients	Retrospective cohort study	RPM-APD (*N* = 65) *N* = 63 used for propensity score matching Mean duration = 0.76 ± 0.27 years	APD without RPM (*N* = 295). N = 63 used for propensity score matching	0.86 + − 0.27y in APD-RPM vs 0.74 + − 0.34y in APD without RPM	Hospitalizations	Less in intervention group (42.6% vs 68.1%, *p* = 0.029)	Low
							Number of hospital days	Less in intervention group (5.59 vs 12.16 days per patients-year, *p* = 0.028)	
Harrington 2014 [29]	USA	N = 6 Age 52.2 ± 6.5y M = 50% CAPD	Pilot study	A tablet computer application allowing real-time monitoring and two-way communication Mean duration = 92 days, SD = not reported	No comparison	8 months	Patient satisfaction (Likert scale (1-10))	5.2 on Likert scale	Moderate
Milan- Manani 2020 [21]	Italy	*N* = 73 Age 60,4 [47.4–75.1] y M: 77% in intervention group; 71% in control group APD	Retrospective cohort study	APD-RM (*N* = 35)	APD standard care (*N* = 38)	6 months	QoL (KDQOL-SF)	N.S. difference	Moderate
							Peritonitis	N.S. difference	
							Transfer to HD (duration not specified)	0 in intervention group, 1 in control group	
							Hospitalizations	N.S. difference in all-causeLess disease-specific hospitalizations in the intervention group (18.2% vs 77.8%, p = 0.022)	
							Frequency of visits	N.S. difference in all-cause (*p* = 0.095)Less urgent visits due to overhydration (*p* = 0.042)	
Dey 2016 [35]	UK	N = 22 Age 61.6 [IQR 26.4–93.4] y M = 55% APD	Pilot study	Computer tablets (PODs) with integrated software for weighing scales and blood pressure machines; patient vital data recording; questionnaire regarding complaints (at beginning and end of study); twice-weekly dietary questionnaire; access to medical and educational information. Mean duration = 341.9 days, SD = not reported	Pre-intervention with PODs	15 months	Quality of life (KDQOL-36)	N.S. difference	Serious
							Patient satisfaction (QUEST)	N.S. difference	
Chaudhuri 2020 [31]	U.S.A.	*N* = 6343 Age 56. 9 ± 15.2y M = 57% % CAPD not specified	Retrospective study	RTM ‘PatientHub’ moderate users (*N* = 673) frequent users (*N* = 1577) RTM involves patients viewing their dialysis orders, laboratory results, medications, supply orders and documenting their daily PD treatment data, vital signs, complications	RTM non-users (*N* = 4093)	12 months	Transfer to HD (> 6wks)	Lower in frequent users versus non-users (p = 0.001, on average 30.5 ± 2.5% lower)	Moderate
							Hospitalizations	Lower in frequent users versus non-users (on average 23.75 ± 1.71% lower, *p* ≤ 0.001)	
							Number of hospital days	Lower in frequent users versus non-users (on average 34.75 ± 2.5% lower, p ≤ 0.001)	
Corzo 2020 [34]	Colombia	*N* = 558 Age 53.8 ± 16.9y M = 60%, APD	Retrospective, multicenter, observational cohort study	APD-RPM (*N* = 148)	APD without RPM (*N* = 410) *N* = 148 used for propensity score matching	1.1 ± 0.6 years	Transfer to HD (>30d)	Lower in intervention group (p = 0.03)	Moderate
							Mortality	N.S. difference(only reported for the non-matched population)	
Nayak 2012 [17]	India	N = 246 Age 51.5 ± 12.8y in rural group 52.3 ± 12.6y in urban group M: 70% in rural group; 69% in urban group %CAPD not specified	Observational	Internet-based RM system (including online log of dialysis data, pictures, access to laboratory results, health records and prescriptions, possibility to schedule appointments and to receive alerts) in rural patients (*N* = 115)	Internet-based RM system (including online log of dialysis data, pictures, access to laboratory results, health records and prescriptions, possibility to schedule appointments and to receive alerts) in urban patients (*N* = 131)	2008 patient-months in the rural group; 2288 patient-months in the urban group	Peritonitis	N.S. difference	Moderate
							Exit-site infection	N.S. difference	
Bunch 2020 [19]	Colombia	*N* = 1.023 Age 63 [IQR 51–72] y M = 61% APD	Observational cohort study	RPM-APD during pandemic (on-site evaluation only for special indications, weekly telephonic triage, daily review APD treatments, technique review through videos sent by patients)	RPM-APD before the covid-19 pandemic (track patient’s adherence, blood pressure, ultrafiltration, and weight daily; perform proactive telephone interventions anticipating possible urgent care requirements)	3 months	Peritonitis	N.S. difference	Serious
							On-site evaluations perpatient/month	Lower in the intervention group *p* < 0.01 (the absolute number of evaluations was not reported)	
							Teleconsultations per patient/month	Higher in the intervention group *p* < 0.01 (the absolute number of teleconsultations was not reported)	
Polanco 2020 [16]	Dominican Republic	N = 913 Age 51 [IQR 19–96] y M = 62% 99.6% CAPD	Observational prospective study	Telemedicine-facilitated PD protocol (monthly telephone contact, psychological and nutritional surveys, pictures of daily dialysis records and lower limbs (possible edema) through Whatsapp if internet was available). Duration = 3 months	Standard PD protocol 3 months prior to implementation of intervention	3 months	Transfer to HD (duration not specified)	N.S. difference	Serious
							Peritonitis	N.S. difference	
							Hospitalizations	N.S. difference	
Viglino 2020 [18]	Italy	*N* = 107 Age 72.2 ± 13.1y M = 59% %CAPD not specified	Observational study	VideoDialysis assisted PD (N = 15) Mean duration = 19.0 ± 12.9 months	Traditional assisted PD (*N* = 62) and self-PD (*N* = 30)	285 months/1869 patient-months	Peritonitis	N.S. difference	Serious
							Time free from first peritonitis	N.S. difference	
							Transfer to HD (duration not specified)	N = 3 (20%) in intervention group versus 17 (18%) in the control group (no statistical analysis performed)	
Lew 2019 [15]	U.S.A.	*N* = 125 Age 56 [IQR 43.6–64.3] y M = 57% < 10% CAPD	Pilot observational study	RBM of weight and bloodpressure and two-way videoconferencing between patient and nurse (*n* = 125) Duration not reported	Costs pre-intervention	No information	Overall costs of care	N.S. difference for overall costs	Serious
								Outpatient visit claim payment amounts decreased post-intervention relative to pre-intervention for those at age 18–54 years. (*p* = 0.0155) In other subgroups (gender, race) non- or nearly significant changes were found.	
							Hospitalizations and length of hospitalization	Less for RBM-collected weight and higher for RBM-collected blood pressure (number of events and length not reported)	
Milan- Manani 2019 [20]	Italy	*N* = 85 Age 56.5 ± 15.5y M = 75% APD	Observational cohort study	RM-APD (*N* = 43) Duration = at least 12 months	Patients with APD without RM (historical cohort) (*N* = 42)	13.28 [IQR 6.65–14.65] months in the invention group 12 months (fixed) in the control group	Hospital savings	€9130 for personnel and €5810 for logistics (*p* < 0.01)	Serious
							In-person visits	Lower in the intervention group (3.56 vs 5.14 visits per patient/year, p < 0.01)	
Dey 2016 [35]	UK	N = 22 Age 61.6 [IQR 26.4–93.4] y M = 55% APD	Pilot study	Computer tablets (PODs) with integrated software for weighing scales and blood pressure machines; patient vital data recording; questionnaire regarding complaints (at beginning and end of study); twice-weekly dietary questionnaire; access to medical and educational information. Mean duration = 341.9 days, SD = not reported	Pre-intervention with PODs	15 months	Quality of life (KDQOL-36)	N.S. difference	Serious
							Patient satisfaction (QUEST	N.S. difference	
Kiberd 2014 [32]	Canada	N = 17 Age 57.1 ± 1.9y M = 52% %CAPD not specified	Pilot study	Web-based portal allowing communication between patients and healthcare team; Duration = 12 months	Pre-intervention	6 and 12 months	Quality of life (CQI and EQ-5D)	N.S. difference as compared to baseline	Critical
							Patient satisfaction (Likert scale (1-10) modified from) [38]	6.5 ± 0.6 on Likert scale	
Magnus 2017 [33]	USA	N = 200 Mean age = not reported M = 51% % CAPD not specified	Observational study	RBM of blood pressure, weight and glucose (if diabetic), including video chat with the healthcare team; access to online educational resources. Duration = not reported	Pre-intervention with RBM; video-chat and/or access to online educational videos	Not reported	Patient satisfaction (26-item TSUQ) [39]	Number of persons that were satisfied or completely satisfied (90.7%) was higher than at baseline (*p* < 0.001)	Critical
							Exit-site infection	10.5% post-intervention and 7.3% pre-intervention (no statistical analysis)	
							Hospitalizations	20.8% pre-intervention and 15.1% post-intervention (no statistical analysis)	

Details and abbreviations Table 1: Age is described as mean age ± standard deviation, if not specified otherwise. *APD* Automated peritoneal dialysis, *CAPD* Continuous ambulatory peritoneal dialysis, *PD* Peritoneal dialysis, *CQI* Consumer quality index, *EQ-5D* EuroQol Five Dimensions Questionnaire, *F* Female, *HD* Hemodialysis, *KDQOL-SF* Kidney Disease Quality of Life Short Form, *KDQOL-36* Kidney Disease Quality of Life − 36 Form, *M* Male, *N* Number of patients, *NS* Non-significant, *SD* Standard deviation, *IQR* Interquartile range, *QoL* Quality of life, *QUEST* Quebec User Evaluation of Satisfaction with assistive Technology, *RBM* Remote biometric monitoring, *RCT* Randomized controlled trial, *RM* Remote monitoring, *RPM* Remote patient monitoring, *RTM* Remote treatment monitoring, *RM-APD* Remote monitoring automated peritoneal dialysis, *SMS* Short messaging service, *y* years, *TSUQ* Telemedicine Satisfaction and Usefulness Questionnaire

### Telehealth interventions

Five studies investigated remote monitoring (RM) during predominantly APD as an intervention [20, 21, 29, 34, 36]. Other studies investigated the implementation of RM, including the possibility to: i. contact the health care team through video-chat [15, 19, 33]; ii. send pictures, view healthcare-records and schedule appointments [17], iii. View laboratory results, medication prescriptions and supply orders [31], iv. access medical information and fill-out online questionnaires [35] (Table 2).

**Table 2:** Overview of included articles grouped by the type of telemedicine interventions and outcomes

**Remote monitoring (RM)**				
**Study**	**Intervention**	**Outcomes**	**Results**	**Risk of bias**
**Quality of Life**				
**Harrington 2014** [29]	RM-CAPD N = 6	Patient satisfaction	5.2 on Likert scale (1-10)	Moderate
**SONG-PD clinical outcomes**				
**Milan-Manani 2020** [21]	RM-APD *N* = 35	Peritonitis Transfer to HD (duration not specified)	N.S. difference 0 in intervention group, 1 in control group	Moderate
**Corzo 2020** [34]	RPM-APD N = 148	Transfer to HD (>30d)	Lower in intervention group (p = 0.03)	Moderate
		Mortality	N.S. difference, only reported for the non-matched population	
**Cost-effectiveness**				
**Milan-Manani 2019** [20]	RM-APD N = 43	Hospital savings	€9130 for personnel and €5810 for logistics (*p* < 0.01)	Serious
**Hospitalizations and health-care consumption**				
**Sanabria 2019** [36]	RPM-APD *N* = 65	Hospitalizations	Less in intervention group (p = 0.029)	Low
		Number of hospital days	Less in intervention group (*p* = 0.028)	
**Milan-Manani 2020** [21]	RM-APD N = 35	Hospitalizations	N.S. difference in all-cause Less disease-specific hospitalizations in intervention group (*p* = 0.022)	Moderate
		Frequency of visits	N.S. difference in all-causeLess urgent visits due to overhydration (*p* = 0.042)	
**Milan-Manani 2019** [20]	RM-APD N = 43	In-person visits	Lower in the intervention group (p < 0.01)	Serious
**Remote monitoring (RM) with additional features**				
**Quality of Life**				
**Dey 2016** [35]	RM-APD + access to medical data and online questionnaires *N* = 22	Quality of life (KDQOL-36)	N.S. difference	Serious
		Patient satisfaction (QUEST)	N.S. difference	
**Magnus 2017** [33]	RBM-APD +videochat and access to educational material *N* = 200	Patient satisfaction	80.1% of participants were either satisfied or completely satisfied with the intervention	Critical
**SONG-PD clinical outcomes**				
**Chaudhuri 2020** [31]	RM-APD + viewing laboratory results, medication prescriptions, supply orders*N* = 2284	Transfer to HD (>6wks)	Lower in frequent users versus non-users (p = 0.001)	Moderate
**Nayak 2012** [17]	RM-APD + send pictures, view healthcare-records and schedule appointments *N* = 246	Peritonitis	N.S. difference	Moderate
		Exit-site infection	N.S. difference	
**Bunch 2020** [19]	RPM-APD + videochat *N* = 1023	Peritonitis rates	N.S. difference	Serious
**Magnus 2017** [33]	RBM-APD + videochat and access to educational material N = 200	Exit-site infections	10.5% post-intervention and 7.3% pre-intervention (no statistical analysis	Critical
**Cost-effectiveness**				
**Lew 2019** [15]	RPM-APD + videochat *N* = 125	Overall costs of care	N.S. difference (except for in certain subgroups)	Serious
**Hospitalizations and health-care consumption**				
**Chaudhuri 2020** [31]	RM-APD + viewing laboratory results, medication prescriptions, supply orders N = 2284	Hospitalizations	Lower in frequent users versus non-users (*p* ≤ 0.001)	Moderate
		Number of hospital days	Lower in frequent users versus non-users (*p* ≤ 0.001)	
**Lew 2019** [15]	RPM-APD + videochat N = 125	Hospitalizations and length of hospitalization	Less for RBM-collected weight and higher for RBM-collected blood pressure	Serious
**Bunch 2020** [19]	RPM-APD + videochat N = 1023	Teleconsultations	Higher in the intervention group (p < 0.01)	Serious
		On site evaluations	Lower in the intervention group (*p* < 0.01)	
**Magnus 2017** [33]	RBM-APD + videochat and access to educational material N = 200	Hospitalizations	20.8% pre-intervention and 15.1% post-intervention (no statistical analysis)	Critical
**Online bi-directional communication between patients and healthcare team**				
**Quality of Life**				
**Cao 2018** [28]	Internet-based instant messaging N = 80	Patient-satisfaction	Higher in the intervention group (p < 0.001)	Unclear
**Li 2014** [30]	Post-discharge nurse-led telephone support N = 69	QoL (KDQOL-SF)	N.S. difference	Unclear
		Patient satisfaction	N.S. difference	
**Kiberd 2018** [32]	Online communication between patient and healthcare team via web-based portal *N* = 17	Quality of life (CQI and EQ-5D)	N.S. difference as compared to baseline	Critical
		Patient satisfaction (Likert scale (1-10))	6.5 on Likert-type scale	
**SONG-PD clinical outcomes**				
**Cao 2018** [28]	Internet-based instant messaging *N* = 80	Exit-site infection	N.S. difference	Unclear
		Peritonitis	Higher in intervention group (60 cases in 80 patients (75%) vs 40 cases in 80 patients (50%) statistical significance not reported)	
		Mortality	Lower in intervention group (*p* = 0.058)	
		Transfer to HD (was not a pre-specified outcome)	N.S. difference	
**Li 2014** [30]	Post-discharge nurse-led telephone support *N* = 69	Peritonitis	N.S. difference	Unclear
		Catheter-infections	N.S. difference	
**Polanco 2020** [16]	Telemedicine-facilitated PD protocol (daily transfer of dialysis records and pictures, monthly contact by telephone *N* = 913	Transfer to HD (duration not specified)	N.S. difference	Serious
		Peritonitis	N.S. difference	
**Viglino 2020** [18]	Video-assisted PD N = 15	Peritonitis	N.S. difference	Serious
		Time free from first peritonitis	N.S. difference	
		Transfer to HD (duration not specified)	N = 3 (20%) in intervention group versus 17(18%) in the control group (no statistical analysis performed)	
**Hospitalizations and health-care consumption**				
**Cao 2018** [28]	Internet-based instant messaging N = 80	Hospitalizations	N.S. difference	Unclear
**Li 2014** [30]	Post-discharge nurse-led telephone support N = 69	Readmissions	N.S. difference	Unclear
		Clinical visits	Less in intervention group (71% vs 47%, p = 0.039)	
**Polanco 2020** [16]	Telemedicine-facilitated PD protocol (daily transfer of dialysis records and pictures, monthly contact by telephone *N* = 913	Hospitalizations	N.S. difference	Serious

*RM* Remote monitoring, *RBM* Remote biometric monitoring, *RM-APD* Remote monitoring automated peritoneal dialysis, *HD* Hemodialysis, *N* Number of patients, *KDQOL-36*, *QoL* Quality of life, *QUEST* Quebec User Evaluation of Satisfaction with assistive Technology Kidney Disease Quality of Life −36 Form, *CQI* Consumer quality index, *EQ-5D* EuroQol Five Dimensions, *KDQOL-SF* Kidney Disease Quality of Life Short Form Questionnaire

The remaining studies investigated a diversity of telehealth interventions aimed at online communication between the patient and the healthcare team, including: internet-based instant messaging software [28]; an eHealth portal software using a web-based application [32]; a nurse-led post-discharge telephone support service [30]; a telemedicine system using video-assisted dialysis (VD) [18] and a telemedicine-facilitated PD protocol, including daily transfer of dialysis records, pictures of lower limbs and monthly contact by telephone [16] (Table 2).

### Risk of bias

The risk of bias of the two included RCTs [28, 30] was classified as unclear, due to uncertainty regarding possible selection and detection bias (Supplementary Material II). The risk of bias of the 14 non-randomized studies was classified as low in one study [36], moderate in five studies [17, 21, 29, 31, 34], serious in six studies [15, 16, 18–20, 35] and critical in two studies [32, 33] (Supplementary Material III).

### Reported outcomes

Of the sixteen included studies, four reported on quality of life [21, 30, 32, 35]. Six studies evaluated patient-satisfaction [28–30, 32, 33]. Clinical outcomes were assessed in ten of the sixteen included studies [16–19, 21, 28, 30, 31, 33, 34]. Of these, six investigated peritonitis rates [18, 19, 21, 28, 30] and in four studies exit-site or catheter infections [17, 28, 30, 33] were evaluated. Technique failure as defined by transfer to HD was reported by six studies [16, 18, 21, 28, 31, 34] and two studies [28, 34] investigated mortality. There were no studies reporting cardiovascular events as a study outcome.

Furthermore, cost-effectiveness was investigated as primary outcome measure by two studies [15, 21]. The number of hospitalizations was studied in eight studies [15, 16, 21, 28, 30, 31, 33, 36], length of hospitalization in three studies [15, 31, 36] and four studies evaluated the number of patient-visits [19–21, 30, 36]. Results are shown in Table 1.

### Quality of life (QoL)

#### QoL

The impact of telehealth interventions on QoL was evaluated in four of the included studies [21, 30, 32, 35], encompassing a total number of 247 patients, with an average age of 58.9 ± 2.6 years. Fifty-five percent of these patients were treated with CAPD [30]. Follow-up ranged from 12 weeks to 15 months in these studies. Both the telehealth interventions and the tools to assess QoL differed among the four studies [21, 30, 32, 35].

#### QoL – RM – studies

One study evaluated RM-APD [21] and another RM-APD with additional features, such as access to medical data and the use of online questionnaires [35]. QoL was assessed using the Kidney Disease Quality of Life Short Form (KDQOL-SF) [21] and by the Kidney Disease Quality of Life − 36 Form (KDQOL-36), respectively [35]. No significant improvement in QoL was observed in either study.

#### QoL – patient communication – studies

The KDQOL-SF was also used in the randomized study by Li et al [30], which investigated a post-discharge nurse-led telephone support service to patients treated with CAPD. Kiberd *et al* [32] evaluated a web-based intervention to facilitate bi-directional communication between PD-patients and healthcare team. In that study, QoL was assessed by use of the Consumer quality index (CQI) and the EuroQol Five Dimensions Questionnaire (EQ-5D) [32]. As in the other studies, no significant improvement in QoL was observed (Table 1).

### Patient satisfaction

The impact of telehealth interventions on patient satisfaction was studied in six of the included reports [28–30, 32, 33, 35]. These comprise a total number of 540 patients, with an average age of 55.9 ± 3.9 years. At least 55.7% of patients were treated with CAPD. Follow-up ranged from 12 weeks to 15 months and types of telehealth intervention differed across the studies (Table 1).

#### Patient satisfaction – RM – studies

Three studies assessed RM-CAPD [29], RM-APD with additional features [35] and remote biometric monitoring (RBM) of blood pressure and weight, with additional features such as video-chat with the healthcare team and access to online educational resources in either CAPD or APD treated patients [33], respectively. Patient satisfaction was investigated by the following tools: the Likert scale at the end of follow-up [29], the Quebec User Evaluation of Satisfaction with assistive Technology questionnaire (QUEST) at the start and end of the follow-up period [35] and by quarterly surveys using the 26-item Telemedicine Satisfaction and Usefulness Questionnaire (TSUQ) [33]. The study by Magnus *et al* [33], involving 200 patients, was the only study that reported significant improvement in patient satisfaction after introduction of RBM. In that study, PD-modality and follow-up time were not specified. The study [33] was considered at critical risk of bias (Supplementary Material III).

#### Patient satisfaction – patient communication – studies

The studied types of telehealth-interventions in the three included studies involved: an internet-based instant messaging service [28], a post-discharge nurse-led telephone support service [30] and an online communication platform via a web-based portal [32].

Tools to assess patient satisfaction differed across the studies [28, 30, 32]. Kiberd et al [32] assessed patient satisfaction using a Likert scale. In the other studies [28, 30] tools for assessing patient satisfaction were not specified. The two randomized studies [28, 30] found a significant improvement in patient satisfaction after introduction of an internet-based messaging service [28] and a post-discharge nurse-led telephone support [30], respectively (Table 1). These studies involved 55% of the total number of patients in which patient satisfaction was evaluated and included 295 patients treated with CAPD [28, 30]. These studies [28, 30] were considered to carry an unclear risk of bias (Supplementary Material II).

### Clinical outcomes

#### PD-related infections

Eight studies evaluated the association between telehealth interventions and peritonitis rate. These studies include a total number of 2857 patients, with an average age of 58.1 ± 7.7 years [16–19, 21, 28, 30, 33]. At least 45.5% of those patients were treated with CAPD (Table 1) [16, 28, 30].

#### PD-related infections – RM – studies

Four of the eight studies investigated RM [17, 19, 21, 33], involving a total number of 1542 patients. A minority (16%) was treated with CAPD. In the study by Nayak et al [17], RM also included several additional features, such as online log of dialysis data and pictures, access to laboratory results, health records and prescriptions, possibility to schedule appointments and to receive alerts [17]. PD-modality was not specified in that study [17]. None of these studies reported significant differences in peritonitis rate after introduction of RM (Table 1).

Exit-site infection rates were reported in two of the studies [17, 33], but no significant associations with the intervention were found (Table 1). In the study by Magnus *et al* [33], involving 200 patients treated with APD, a higher number of exit-site infections were reported post-intervention (10.5%), as compared to pre-intervention (7.3%) [33]. No statistical analysis was performed in that study.

#### PD-related infections – patient communication – studies

In the four remaining studies [16, 18, 28, 30] involving PD-related infections, the following telehealth interventions were investigated: videodialysis-assisted PD [18], an internet-based instant messaging service [28], a post-discharge nurse-led telephone support service [30] and a telemedicine-facilitated PD protocol with bi-directional contact between patient and healthcare team [16]. PD-modality was not specified in the study by Viglino et al. [18].

One study reported a significantly higher peritonitis rate after introduction of the telehealth intervention [28]. In the study by Cao et al [28], involving 160 patients with a follow-up time of 11.4 ± 1.5 months a peritonitis rate of 60 episodes was found in the group that used an internet-based instant messaging service, as compared to 40 in the control group. Statistical significance was not reported (Table 1).

Exit-site infection rate was reported in two studies [28, 30]. No significant associations with the telehealth interventions were found (Table 1).

### Mortality

Two studies [28, 34] reported associations between telehealth interventions and mortality. The study by Cao *et al* [28] evaluated an internet based instant messaging service in 80 CAPD-treated patients as compared to 80 controls without this service, with a follow-up time of 11.4 ± 1.5 months [28]. These authors found a lower mortality in the intervention group as compared to the control group (*p* = 0.058), yet the number of events in each group was not reported [28]. That study [28] was considered to carry an unclear risk of bias (Supplementary Material II).

Corzo *et al* [34] reported no significant differences in mortality (Table 1).

### Transfer to HD

Six studies evaluated associations between telehealth interventions and transfer to hemodialysis [16, 21, 28, 31, 34, 40]. These studies comprise a total of 8054 participants, with an average age of 58.6 ± 7.2 years. At least 13.3% of patients were treated with CAPD (Table 1). The duration of HD in the definition of this outcome was unspecified in most studies, with the exception of the studies by Corzo *et al* [34] and Chaudhuri et al. [31] In these reports, this was defined as hemodialysis for at least 30 days [34] and 6 weeks [31], respectively.

#### Transfer to HD – RM – studies

The association of RM-APD with transfer to HD was investigated in three studies [21, 31, 34], of which one studied RM-APD with additional features [31]. In the largest study included in this review, accounting for 78% of the total number of participants, transfer to HD was significantly lower in the 1586 frequent RM-APD users as compared to the 4123 non-users, evaluated after 12 months follow-up (*p* = 0.001) [16]. Furthermore, in the study by Corzo et al [34], a significant reduction in transfer to HD was found in 148 patients who had used RM-APD, as compared to 148 propensity-matched controls (*p* = 0.03), after a mean follow-up time of 1.1 ± 0.6 years. Milan-Manani et al [21] investigated RM-APD in 73 participants and found no transfers to HD after 6 months in the intervention group (*N* = 35), as compared to one patient in the control group (*N* = 38). These three studies [21, 31, 34] were considered to carry a moderate risk of bias (Supplementary Table III).

#### Transfer to HD – patient communication – studies

The three remaining studies [16, 18, 28] involving transfer to HD investigated the following telehealth interventions: an internet-based instant messaging software system [28], a telemedicine-facilitated PD protocol [16] and a video dialysis system [18]. In the study by Viglino et al [18], evaluating video-assisted PD in 15 patients, as compared to 92 controls with either traditionally assisted PD or self-PD, three (20%) transfers to HD were reported, as compared to seventeen (18%) in the control group (Table 1). That study [18] was considered at serious risk of bias (Supplementary Material III). The remaining two studies investigating transfer to HD [16, 28] did not report any differences as compared to the control group (Table 1).

### Cost-effectiveness

Two studies evaluated the association of telehealth interventions with cost-effectiveness [15, 20]. The study by Milan-Manani et al [20] evaluated RM-APD in 43 patients, as compared to 42 patients without RM from a historical cohort. They found a significant increase in hospital savings in terms of costs for personnel and logistics 12 months after introduction of RM-APD (Table 1) [20]. In the study by Lew et al [15], overall costs of care were reduced after introduction of RBM of weight and blood pressure and two-way videoconferencing between patient and nurse in 125 patients, as compared to standard care without daily RBM. Duration of the intervention and follow-up time was not specified in the latter study (Table 1) [15]. These two studies were considered to carry a serious risk of bias (Supplementary material III) [15, 20].

### Secondary outcomes

#### Hospitalizations

Associations between telehealth interventions and hospitalization rates were evaluated in eight of the included studies (Table 1) [15, 16, 21, 28, 30, 31, 33, 36]. These reports encompass a total of 8309 patients, with an average age of 55.7 ± 3.2 years. Of these patients, at least 14.5% were treated with CAPD. Average follow-up was 7.6 ± 4.1 months.

#### Hospitalizations – RM – studies

RM-(A)PD was studied in five studies [15, 21, 31, 33, 36], three of which included RM-(A)PD with additional features [15, 31, 33]. Of these five studies (total *N* = 7101), three reported significantly lower hospitalization rates after introduction of the telehealth interventions (Table 1) [21, 31, 36]. In the study by Sanabria et al [36], hospitalizations were significantly lower in 63 patients with RPM-APD as compared to 63 propensity-matched controls without RPM-APD (*p* = 0.028). In the report by Chaudhuri *et al* [31], hospitalization rates after 12 months were significantly lower in the 1586 frequent users of the remote treatment monitoring (RTM) intervention (Table 1), as compared to the 4123 non-users in that study (*p* ≤ 0.001). The study by Milan-Manani *et al* [21] reported a non-significant difference in all-cause hospitalization rate. Yet, a significantly lower disease-specific hospitalization rate was observed after 6 months in 35 patients with RM-APD, as compared to 38 patients without RPM [21]. This was 18.2% in the RM-APD group compared to 77.8% in the control group (*p* = 0.022) [21]. These studies were considered to carry a moderate [21, 31] or low [21] risk of bias, respectively.

#### Hospitalizations – patient communication – studies

The remaining three studies evaluated various types of online bi-directional communication between patients and the healthcare team (Table 2) [16, 28, 30]. No significant associations between the implemented telehealth interventions and hospitalizations were reported.

#### Length of hospitalization

Three studies, involving RM with additional features such as access to laboratory results, medication prescriptions, supply orders [31] and videochat [15], investigated associations between telehealth interventions and length of hospitalization [15, 31, 36].

The retrospective studies by Sanabria *et al* [36] and Chaudhuri *et al* [31] (*N* = 6743, aged 57 ± 0.1 years) reported a significantly reduced length of hospitalization after introduction of the telehealth interventions (Table 1) [31, 36]. In the study by Sanabria *et al* [36], length of hospitalization was 5.59 days per patient-year in 65 patients treated with RPM-APD, as compared to 12.16 days per patient-year in 295 patients without RPM-APD (*p* = 0.028). Chaudhuri *et al* [31] reported an average 34.75 ± 2.5% lower hospital length in frequent users of a RTM-system, as compared to non-users (*p* ≤ 0.001). These studies were considered to carry a low [36] and moderate [31] risk of bias, respectively (Supplementary Material III).

Lew *et al* [15] showed conflicting results with respect to this outcome (Table 1). This latter study was considered to be at serious risk of bias (Supplementary Material III) [15].

#### Number of (in-person) visits

The four studies that evaluated this outcome, all found a significantly lower number of in-person visits after introduction of the telehealth intervention (Table 1) [19–21, 30]. Three of these investigated RM-APD [19–21], of which one with the additional availability of videochat [19]. In the remaining study [30], an online bidirectional communication system was studied in a population treated with CAPD. These studies represent a total of *N* = 1316 patients, with an average age of 59.1 ± 3.3 years. Mean follow-up time was 6.3 ± 4.9 months. Manani *et al* [20] reported a median number of in person visits of four (3.0–5.0) in the RM-APD group, as compared to five (4.25–5.75) in the control group (*p* < 0.01). In another study [21] by the same authors, a lower number of clinic visits was found in patients treated with RM-APD, as compared to the control group (0.17 ± 0.45 versus 0.66 ± 1.36, *p* = 0.042). This was in line with the study by Bunch et al [19], yet the absolute number of events was not reported in that study. Finally, Li et al [30] reported a significantly lower number of clinic visits at the end of follow-up in the intervention group (32 visits in the intervention group as compared to 58 visits in the control group, *p* = 0.039). These studies involved one randomized study with unclear [30] risk of bias (Supplementary Material II) and three observational studies with a moderate [19, 21] and serious [20] risk of bias, respectively (Supplementary Material III).

## Discussion

In this review, we described the current evidence on the clinical and economic benefit of telehealth interventions added to PD care. Despite the growing number of reports on telehealth initiatives in PD, the evidence remains limited. This is due to a large heterogeneity between studies in terms of: study design, type and duration of the telehealth intervention, duration of follow-up, lack of information on adherence in all but one study [21] and the chosen clinical and economic outcomes. Except for two randomized trials [28, 30], all studies were observational and thereby subject to various degrees of risk of bias (Table 1).

Potential sources of bias included: patient characteristics and selection, involving health literacy, education level and/or access to e-health; limited information on loss to follow-up and deviations from intended interventions, as well as handling of missing data. Nevertheless, the included recent studies indicate that RPM might reduce transfer to hemodialysis, as well as healthcare consumption.

A similar review on e-health interventions in PD care was recently published by others [14]. That review included 15 studies, published between 1992 and 2018, representing 1343 patients receiving PD. SONG-PD outcomes were evaluated as primary outcomes, as well as hospitalization rates [14]. As compared to that report, this review included 16 more contemporary studies published between 2012 and 2020, representing an 8-fold larger PD-treated population (*N* = 10,373). This allowed a first review of associations between telehealth interventions and transfer to HD. This outcome of interest could not be evaluated previously [14]. Our current findings indicate a potential benefit of RPM in terms of PD-technique survival. This is an important finding that warrants further investigation. Furthermore, in the current review associations of telehealth interventions with healthcare resource consumption could be evaluated into greater extent than previously reported [14]. Based on our synthesis, it can be argued that telehealth interventions, and RPM in particular, could potentially reduce hospitalization rates, as well as healthcare resource consumption in terms of hospitalization length and the number of in-person visits. This is consistent with several other reports in which RM-APD was evaluated [40–42]. These reports were excluded from this review, because these concerned simulation studies. Hence, telehealth interventions in PD may induce favorable economic impact. However, this remains to be established, as at present cost-effectiveness of telehealth interventions in PD care has only been evaluated in two relatively small-scaled studies, with a serious risk of bias [15, 20]. In the previous review by Cartwright *et al* [14], economic impact could be evaluated only in one study with 125 participants and a critical risk of bias. Finally, in line with the previous review [14], we report mixed results on the other outcomes of interest, such as PD-related infections, mortality and QoL.

At present, ‘telehealth’ is a catch-all term for a large variety of interventions in which digital applications are used in healthcare. This is reflected by the large diversity of tools used throughout the studies included in this review. RM-APD is the intervention most extensively studied in PD care thus far. Less is known regarding the benefit of telehealth interventions in the CAPD-population, as patients treated with CAPD (*N* = 1213) comprised merely 11% of the total number of patients in the studies included in this review. This is an issue to address in future studies, as CAPD is used more frequently than APD in many parts of the world [43].

Moreover, in the included studies, there is hardly any information regarding the arguments supporting the choice of a specific telehealth intervention in a specific PD-population. Before one can truly evaluate clinical and economic benefit of telehealth intervention, it is important to investigate user needs and preferences, adoption, user satisfaction and compliance in the specific patient population first [44]. This applies to both patients and caregivers as users of the telehealth tools. In addition, prior to engaging in outcome studies, it is important to investigate and to overcome possible barriers to the use of and access to telehealth, such as socio-economic or language barriers, as well as health illiteracy [37]. This would not only aid to define the best telehealth intervention to study but would also reduce risk of bias in the outcome studied. Finally, it is important to timely address possible health-service barriers, such as integration of the applications into electronic patient charts and the concomitant cybersecurity risks and privacy legislation [37].

## Conclusions

Altogether, there is a need for high-quality, adequately powered prospective trials to assess the clinical and economic benefit of telehealth interventions in PD. Prior to designing those studies, we emphasize consensus on the type of telehealth-interventions, based on user acceptance and feasibility data in the specific PD population, including patients treated with CAPD. This might reduce variability in the interventions and this in turn can increase generalizability. Furthermore, future studies should investigate whether telehealth interventions can be valuable as a surrogate for, rather than an addition to, standard PD-care, especially considering the risk of future pandemics.

Finally, we advocate the use of SONG-PD outcomes [23] in further studies, including life participation and cardiovascular disease, since those outcomes have not yet been studied in this respect. An interesting initiative in this respect is the currently ongoing prospective PDTAP study [45]. Yet, additional randomized studies are warranted.

## Supplementary Information

**Additional file 1.**

## Data Availability

All data generated or analysed during this study are included in this published article and its Supplementary file. This review has not been registered.
