# Supplementary material for: Impact of telehealth interventions added to peritoneal dialysis-care: a systematic review
Source: BMC Nephrol. 2022 Aug 23;23:292. doi: 10.1186/s12882-022-02869-6 (PMC9396599; doi:10.1186/s12882-022-02869-6)
Supplement: Supplementary file 1 — Additional file 1. [file 12882_2022_2869_MOESM1_ESM.docx]

**SUPPLEMENTARY MATERIAL**

IMPACT OF TELEHEALTH INTERVENTIONS ADDED TO PERITONEAL DIALYSIS-CARE: a systematic review

Geertje K.M. Biebuyck, MSc^1,2^; Carola W.H. de Fijter, MD, PhD^3^; Aegida Neradova, MD, PhD^1,2^; Lily Jakulj MD, PhD^1,2^

^1^ Dianet Dialysis Center Amsterdam; ^2^Department of Internal Medicine and Nephrology, Amsterdam University Medical Center, University of Amsterdam, the Netherlands; ^3^Department of Internal Medicine and Nephrology OLVG, Amsterdam, the Netherlands

**Corresponding author:**

Geertje Biebuyck, MSc

Dianet Dialysis Center/Division of Nephrology, Department of Medicine,

Amsterdam University Medical Center,

Meibergdreef 9 1105 AZ Amsterdam, the Netherlands

T: + 32491302778

E: g.k.biebuyck@amsterdamumc.nl

1. **Search strategy**

1. "peritoneal dialysis"[MeSH Terms] (26532)

2. Dialysis, peritoneal OR intermittent peritoneal dialysis OR peritoneal dialysis OR peritoneal dialysis, intermittent OR peritoneum dialysis (33372)

3. 1 OR 2 (33372)

4. "telemedicine"[MeSH Terms] OR "telehealth"[MeSH Terms] OR "ehealth"[MeSH Terms] (33465)

5. "Tele medicine"[tiab] OR "telemedicine"[tiab] OR "e-health"[tiab] OR "ehealth"[tiab] OR "tele-health"[tiab] OR "telehealth"[tiab] OR "distant monitoring (patient) "[tiab] OR "distant patient monitoring"[tiab] OR "remote monitoring (patient)"[tiab] OR "remote patient monitoring"[tiab] OR "tele monitoring"[tiab] OR "telemonitoring"[tiab] OR "remote treatment monitoring"[tiab] (29140)

6. 4 OR 5 (47118)

7. "cell phone"[MeSH Terms] OR "cell phones"[MeSH Terms] OR "tablets"[MeSH Terms] (41866)

8. "device"[tiab] OR "devices"[tiab] OR "cell phone"[tiab] OR "cell phones"[tiab] OR "cellphone"[tiab] OR "cellphones"[tiab] OR "cellular phone"[tiab] OR "cellular telephone"[tiab] OR "mobile phone"[tiab] OR "mobile telephone"[tiab] OR "tablet"[tiab] OR "tablets"[tiab] OR "smart phone"[tiab] OR "smartphone"[tiab] OR "smartphones"[tiab] OR "device"[tiab] OR "virtual consulation"[tiab] OR "video consultation"[tiab] (521323)

9. 7 OR 8 (533697)

10. 6 OR 9 (572723)

11. 3 AND 10 (582)

12. "2010/01/01"[Date - Publication]: "2021/04/06"[Date - Publication]

13. 11 AND 12 (271)

14. "English"[Language] OR "Dutch"[Language] OR "French"[Language]

15. 13 AND 14

1. **Cochrane Risk of Bias assessment for randomized trials**

***Li 2014***

| Bias domains | Author’s judgment | Support for judgment |
| --- | --- | --- |
| Random sequence generation (selection bias) | Low risk | Patients were assigned to the study or control group using fifty sets of computer-generated random numbers |
| Allocation concealment (selection bias) | Unclear risk | The method used to conceal the allocation sequence is unclear |
| Blinding of participants and researchers  (performance bias) | Unclear risk | It is unclear if and to what extent a participant was aware of the treatment received |
| Blinding of outcome assessment (detection bias) | Unclear risk | It is unclear if and to what extent a participant was aware of the treatment received. |
| Incomplete outcome data (attrition bias) | High risk | The completeness of outcome data for each main outcome is unclear. Attrition and exclusions were not clearly reported. |
| Selective reporting (reporting bias) | Low risk | All pre-specified outcomes were reported |
| Other bias | Unclear risk | No description of adherence which could result in performance bias. |
| Overall risk | Unclear risk | Unclear risk in multiple domains. |

***Cao 2018***

| Bias domains | Author’s judgment | Support for judgment |
| --- | --- | --- |
| Random sequence generation (selection bias) | Low risk | A random number table was used to randomly assign the 160 participants  to the intervention or comparison groups. |
| Allocation concealment (selection bias) | Unclear risk | The method used to conceal the allocation sequence is unclear. |
| Blinding of participants and researchers  (performance bias) | Unclear risk | It is unclear if and to what extent a participant was aware of the treatment |
| Blinding of outcome assessment (detection bias) | Unclear risk | It is unclear if and to what extent a participant was aware of the treatment |
| Incomplete outcome data (attrition bias) | Low risk | The outcome data and losses for each main outcome were reported. |
| Selective reporting (reporting bias) | Low risk | All pre-specified outcomes were reported |
| Other bias | Unclear risk | No description of adherence or number of patients that received additional assistance from caregivers which could result in performance bias |
| Overall risk | Unclear risk | Unclear risk in multiple domains |

1. **ROBINS-1 Risk of Bias assessment for non-randomized studies**

***Bunch 2020***

| Bias domains | Author’s judgment | Support for judgment |
| --- | --- | --- |
| Confounding | Serious | Pandemic itself is likely to predict the outcome of interest |
| Selection of participants | Low | All participants who were likely eligible for the study were likely included |
| Classification of interventions | Low | Intervention is well defined |
| Deviations from intended interventions | No information | Deviations were not reported |
| Missing data | No information | Loss to follow-up, withdrawal or number of patients included in the analysis are not described |
| Measurement of outcomes | Low | Outcome measures were objectively measured |
| Selection of the reported result | No information | The intended outcomes were not pre-specified |
| Overall risk | Serious | The study is judged to be at serious risk of bias in at least one domain, but not at critical  risk of bias in any domain |

***Corzo 2020***

| Bias domains | Author’s judgment | Support for judgment |
| --- | --- | --- |
| Confounding | Moderate | Confounding expected, yet validity and reliability of all important domains was strengthened by propensity-score matching. |
| Selection of participants | Low | Selection of participants was strongly related to the study intervention, yet this was largely overcome by propensity score matching |
| Classification of interventions | Low | Intervention is well defined |
| Deviations from intended interventions | Moderate | Deviations from intended interventions were not sufficiently reported |
| Missing data | No information | There was no report on missing data |
| Measurement of outcomes | Low | Outcome measures were objectively measured |
| Selection of the reported result | Low | All pre-specified outcomes were reported |
| Overall risk | Moderate | There is no clear indication that the study is at serious or critical risk of bias and there is a  lack of information in one or more key domains of bias |

***Chaudhuri 2020***

| Bias domains | Author’s judgment | Support for judgment |
| --- | --- | --- |
| Confounding | Moderate | Confounding expected but adjustment of the analysis for potential confounders was sufficient |
| Selection of participants | Low | All participants who were likely eligible for the trial were likely included in the study |
| Classification of interventions | Low | Intervention is well defined |
| Deviations from intended interventions | Low | Deviations from intended interventions were reported |
| Missing data | Low | Handling of missing data is reported |
| Measurement of outcomes | Low | Outcome measures were objectively measured |
| Selection of the reported result | Low | All pre-specified outcomes were reported |
| Overall risk | Moderate | The study is judged to be at low or moderate risk of bias for all domains |

***Dey 2016***

| Bias domains | Author’s judgment | Support for judgment |
| --- | --- | --- |
| Confounding | Serious | All known important domains are not appropriately controlled for |
| Selection of participants | Serious | Selection of participants was strongly related to the study intervention |
| Classification of interventions | Low | The intervention is well defined |
| Deviations from intended interventions | No information | Deviations were not reported |
| Missing data | No information | Loss to follow-up, withdrawal or number of patients included in the analysis are not described |
| Measurement of outcomes | Low | Validated tools for assessment of QoL and patient satisfaction were used |
| Selection of the reported result | Low | The reported results corresponded to the intended primary outcomes (new data in the discussion section regarding healthcare consumption were not taken into account) |
| Overall risk | Serious | The study is judged to be at serious risk in two domains but not at critical risk in any domain |

***Harrington 2014***

| Bias domains | Author’s judgment | Support for judgment |
| --- | --- | --- |
| Confounding | Moderate | Confounding expected, but validity and reliability of all important domains were sufficient |
| Selection of participants | Low | All participants who were likely eligible for the trial were likely included in the study |
| Classification of interventions | Low | Intervention is well defined |
| Deviations from intended interventions | No information | Deviations were not reported |
| Missing data | Low | Missing data were adequately reported |
| Measurement of outcomes | Moderate | Outcomes were self-reported, unclear whether outcome assessors were aware of intervention status |
| Selection of the reported result | Low | The reported results corresponded to the intended primary outcomes |
| Overall risk | Moderate | The study is judged to be at moderate risk in two domains but not serious in any domain |

***Kiberd 2018***

| Bias domains | Author’s judgment | Support for judgment |
| --- | --- | --- |
| Confounding | Moderate | Confounding expected, but validity and reliability of all important domains were sufficient |
| Selection of participants | Low | All participants who were likely eligible for the trial were likely included in the study |
| Classification of interventions | Low | Intervention is well defined |
| Deviations from intended interventions | No information | Deviations were not reported |
| Missing data | Critical | Data from participants who did not complete the study (approximately 30%) were not reported |
| Measurement of outcomes | Moderate | Outcomes were self-reported, unclear whether outcome assessors were aware of intervention status |
| Selection of the reported result | Low | The reported results corresponded to the intended primary outcomes |
| Overall risk | Critical | The study is judged to be at critical risk in one domain. |

***Lew 2019***

| Bias domains | Author’s judgment | Support for judgment |
| --- | --- | --- |
| Confounding | Serious | All known important domains are not appropriately controlled for |
| Selection of participants | Moderate | Selection of participants may have been related to the study intervention |
| Classification of interventions | Moderate | The intervention is well defined, except for duration of the intervention |
| Deviations from intended interventions | No information | Deviations were not reported |
| Missing data | No information | Missing data were not reported |
| Measurement of outcomes | Moderate | Outcome measures were objectively measured, although duration of follow-up was unclear |
| Selection of the reported result | Low | The reported results corresponded to the intended primary outcomes |
| Overall risk | Serious | The study is judged to be at serious risk in one domain but not at critical risk in any domain |

***Magnus 2017***

| Bias domains | Author’s judgment | Support for judgment |
| --- | --- | --- |
| Confounding | Serious | All known important domains are not appropriately controlled for |
| Selection of participants | Low | All participants who were likely eligible for the trial were likely included in the study |
| Classification of interventions | Critical | Each component  of the intervention was optional and was allowed to be used only once and changed throughout the study period |
| Deviations from intended interventions | No information | Deviations were not reported |
| Missing data | No information | Missing data were not consistently reported |
| Measurement of outcomes | Moderate | Surveys were self-reported |
| Selection of the reported result | Low | The reported results corresponded to the intended primary outcomes |
| Overall risk | Critical | The study is judged to be at critical risk in at least one domain |

***Milan-Manani 2019***

| Bias domains | Author’s judgment | Support for judgment |
| --- | --- | --- |
| Confounding | Serious | All known important domains are not appropriately controlled for |
| Selection of participants | Moderate | Selection of participants may have been related to the study intervention (historical cohort was not matched) |
| Classification of interventions | Low | The intervention is well defined |
| Deviations from intended interventions | Moderate | Deviations from intended interventions were not reported |
| Missing data | No information | Missing data were not reported |
| Measurement of outcomes | Low | Outcome measures were objectively measured |
| Selection of the reported result | Low | All pre-specified outcomes were reported |
| Overall risk | Serious | The study is judged to be at serious risk in one domain but not at critical risk in any domain |

***Milan-Manani 2020***

| Bias domains | Author’s judgment | Support for judgment |
| --- | --- | --- |
| Confounding | Moderate | Confounding expected, but validity and reliability of all important domains were sufficient |
| Selection of participants | Moderate | Deviations from intended interventions were not reported |
| Classification of interventions | Low | The intervention is well defined |
| Deviations from intended interventions | Moderate | Deviations from intended interventions were not reported |
| Missing data | No information | Missing data were not reported |
| Measurement of outcomes | Low | Outcome measures were objectively measured |
| Selection of the reported result | Low | All pre-specified outcomes were reported |
| Overall risk | Moderate | The study is judged to be at low or moderate risk of bias in almost all domains and there is a lack of information in one key domain |

***Nayak 2012***

| Bias domains | Author’s judgment | Support for judgment |
| --- | --- | --- |
| Confounding | Moderate | Confounding expected, but validity and reliability of all important domains were sufficient |
| Selection of participants | Low | All participants who were likely eligible for the trial were likely included in the study |
| Classification of interventions | Low | The intervention is well defined |
| Deviations from intended interventions | No information | Deviations form intended interventions were not reported |
| Missing data | No information | Missing data were not reported |
| Measurement of outcomes | Low | Outcome measures were objectively measured |
| Selection of the reported result | Low | Pre-specified outcomes were reported |
| Overall risk | Moderate | The study is judged to be moderate risk of bias in one domain and there is a lack of information in two key domains. |

***Polanco 2020***

| Bias domains | Author’s judgment | Support for judgment |
| --- | --- | --- |
| Confounding | Serious | Pandemic itself is likely to predict the outcome of interest |
| Selection of participants | Low | All participants who were likely eligible for the trial were likely included in the study |
| Classification of interventions | Low | The intervention is well defined |
| Deviations from intended interventions | No information | Deviations form intended interventions were not reported |
| Missing data | No information | Missing data were not reported |
| Measurement of outcomes | Low | Outcome measures were objectively measured |
| Selection of the reported result | Moderate | Intended outcomes were not pre-specified |
| Overall risk | Serious | The study is judged to be at serious risk in one domain but not at critical risk in any domain |

***Sanabria 2019***

| Bias domains | Author’s judgment | Support for judgment |
| --- | --- | --- |
| Confounding | Low | Confounding expected, yet validity and reliability of all important domains was strengthened by propensity-score matching. |
| Selection of participants | Low | Selection of participants was strongly related to the study intervention, yet this was largely overcome by propensity-score matching |
| Classification of interventions | Low | Intervention is well defined |
| Deviations from intended interventions | Low | Deviations form intended interventions were reported |
| Missing data | No information | There was no report on missing data |
| Measurement of outcomes | Low | Outcome measures were objectively measured |
| Selection of the reported result | Low | All pre-specified outcomes were reported |
| Overall risk | Low | The study is at low risk of bias in almost all domains and there is a lack of information in one key domain |

***Viglino 2020***

| Bias domains | Author’s judgment | Support for judgment |
| --- | --- | --- |
| Confounding | Serious | All known important domains are not appropriately controlled for |
| Selection of participants | Serious | Selection of participants was strongly related to the study intervention |
| Classification of interventions | Low | The intervention is well defined |
| Deviations from intended interventions | Low | Deviations from intended interventions were reported |
| Missing data | No information | Missing data were not reported |
| Measurement of outcomes | Low | Outcome measures were objectively measured |
| Selection of the reported result | Low | All pre-specified outcomes were reported |
| Overall risk | Serious | The study is judged to be at serious risk in two domains but not at critical risk in any domain |
